# Optimising exposure in shoulder arthroplasty: novel use of the Gripper table retraction system

**DOI:** 10.1308/rcsann.2022.0153

**Published:** 2024-02-01

**Authors:** B Amini, R Cuthbert, A Rashid

**Affiliations:** University College London Hospital, UK

## Background

Optimal glenoid exposure is integral to successful shoulder arthroplasty. This is traditionally reliant on surgical assistants maintaining positioning of handheld retractors for long periods in a confined space. Consequently, the experience and availability of surgical assistants impacts directly on operative efficiency.

Use of the Gripper table-mounted retraction system—Gripper (MedEnvision)—to maximise exposure during the anterior approach for hip arthroplasty is commonplace.^1^ Our unit has pioneered use of the Gripper for both primary and revision shoulder arthroplasty.^2^ Each unit costs £110 and is purchased in sets of ten.

## Technique

**Figure 1:**
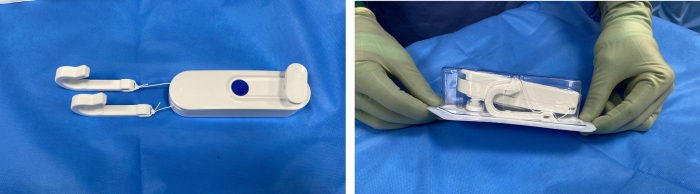
Gripper table-mounted retraction system

**Figure 2:**
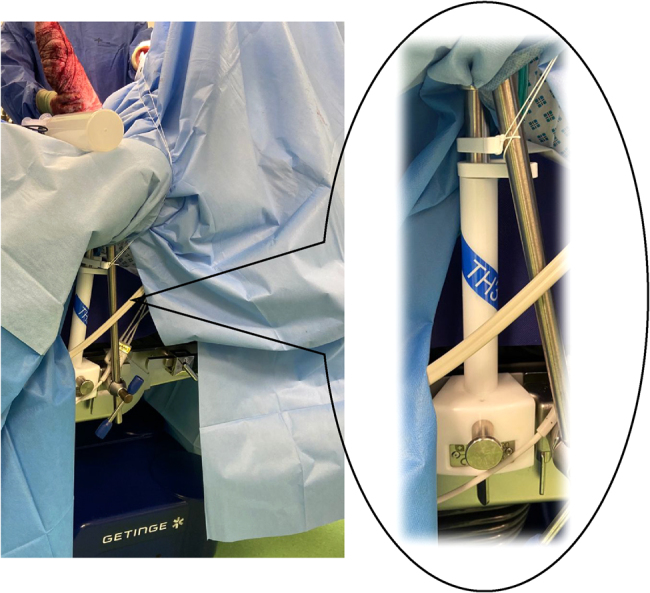
Gripper mounted on operating table

Gripper is a single-use, sterile device comprising a pulley system mounted on the operating table that can be attached to standard flat-handled orthopaedic retractors (Figure 1). Once locked, the pulley system offers continual, untiring retraction with accurate positioning and stable force determined by the lead surgeon (Figure 2).

**Figure 3:**
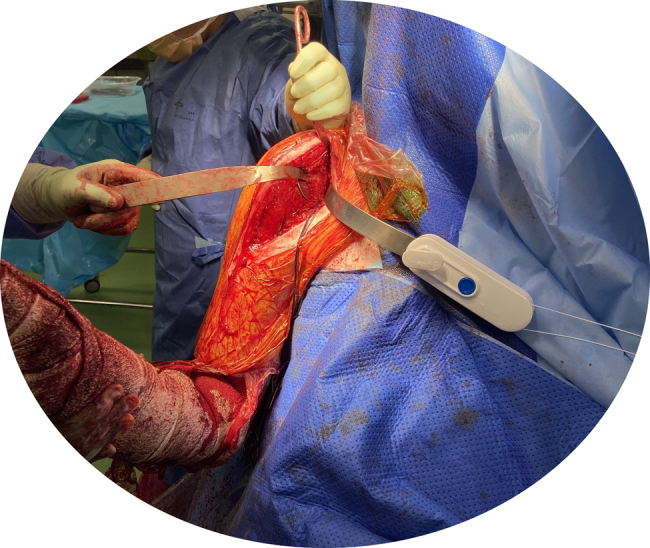
Gripper attached to the anterior glenoid retractor to optimise exposure

Glenoid exposure in shoulder arthroplasty must facilitate unhindered instrumentation to allow correct component positioning.^3^ We recommend attachment of the Gripper to the anterior glenoid retractor (Figure 3). This prevents need for the surgical assistant to reach across the head and body of the draped patient, and allows them to remain on the same side as the lead surgeon.

## Discussion

At our institution, use of the Gripper has enhanced intraoperative ergonomics during shoulder arthroplasty. The Gripper is particularly effective for anterior glenoid exposure where surgical assistants are often required to adopt uncomfortable positions reaching across the patient.

The Gripper is easy to operate, and provides a tireless, consistent assistant while freeing the surgical team to assist the lead surgeon more effectively.
